# The investigation of selective pre-pattern free self-assembled Ge nano-dot formed by excimer laser annealing

**DOI:** 10.1186/1556-276X-7-307

**Published:** 2012-06-18

**Authors:** Min-Hung Lee, Pin-Guang Chen

**Affiliations:** 1 Institute of Electro-Optical Science and Technology, National Taiwan Normal University, Taipei 116, Taiwan

**Keywords:** Re-crystallization, Self-assembled, Excimer laser

## Abstract

Localized Ge nano-dot formation by laser treatment was investigated and discussed in terms of strain distribution. The advantage of this technique is patterning localization of nano-dots without selective epitaxial growth, reducing costs and improving throughput. Self-assembled Ge nano-dots produced by excimer laser annealing statistically distributed dot density and size dependent on laser energy. Improvement in the crystallization quality of the dots was also studied, and a strain analysis was undertaken.

## Background

Self-assembled Ge quantum dots and well arrangements have attracted a lot of attention due to their ability of being integrated into silicon-based optoelectronic and nano-electronic devices [1,2]. One of the motivations behind these efforts is to form devices and functions that take advantage of quantum confinement effects for electronic and optical applications, such as light emitting diodes, tunneling diodes, detectors, etc. [3,4], where Ge quantum dots were formed by ultra-high vacuum chemical vapor deposition (UHV-CVD). Recently, the Ge dots multilayer solar cell made by molecular beam epitaxy was reported [5]. Another motivation is to find cost-effective methods for forming nanoscale devices without using expensive lithography techniques. Among the different ways in producing quantum dots [6-8], excimer laser annealing (ELA) to induce self-assembled Ge islands is an innovative technique. ELA has been widely used in low temperature poly-Si thin film-transistor processes for flat panel display products [9]. One of the characteristics of laser annealing is a shallow absorption depth, which avoids heating of layers underneath, due to the high absorption coefficient of semiconductor material in the UV laser wavelength range [10]. An advantage of laser annealing is the determination of dot localization on the surface. By contrast, dot patterning is difficult to directly use conventional chemical vapor deposition processes, except for selective epitaxial growth (SEG) over pre-patterned samples. Weizman et al. reported the formation of a ripple structure to hillock structure by single laser pulse with silicon-germanium (Si_1−x_Ge_x_), 0.3 < x < 0.7 [11]. In this work, we propose nominal pure Ge re-crystallization and self-assembly into dots by excimer laser irradiation, and discuss the resulting improvement in Ge crystallization quality. We also study the relationship between dot volume and beam energy.

## Methods

**Figure 1:**
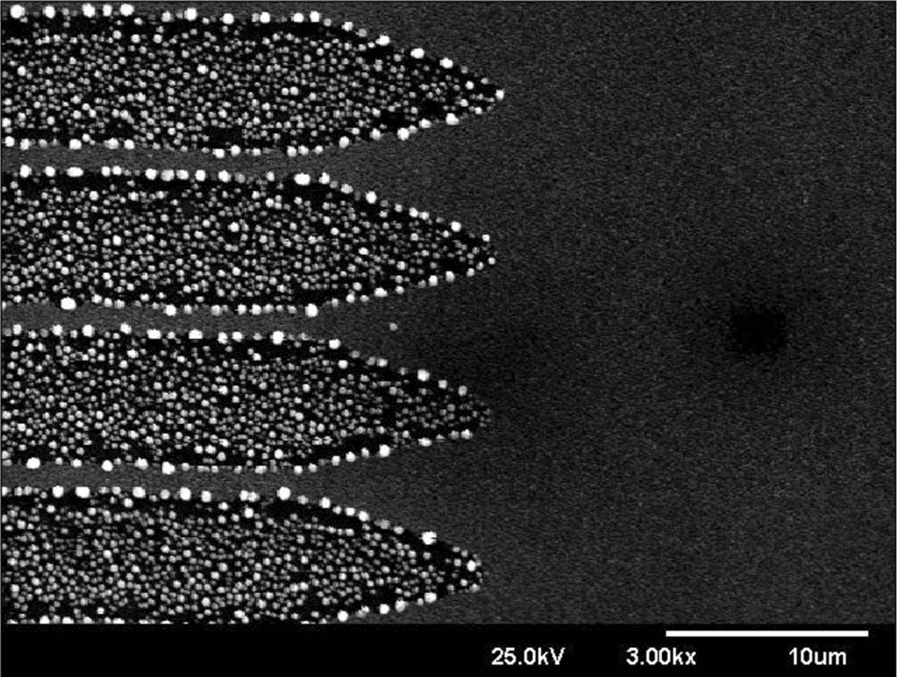
Selective pre-pattern free self-assembled Ge nano-dots were locally formatted by laser annealing technique.

**Figure 2:**
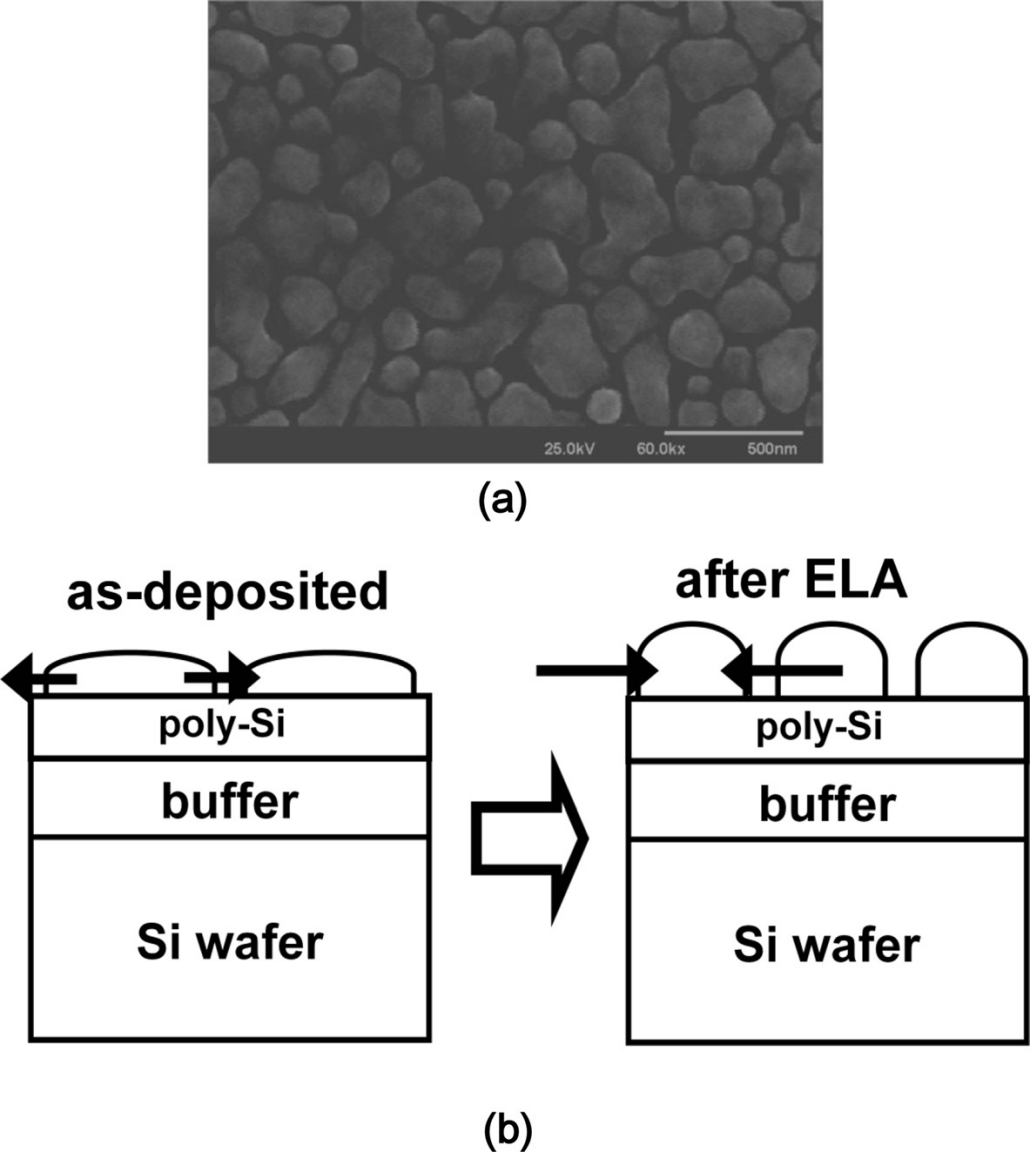
**Top-view SEM of as-deposited poly Ge by UHV-CVD (a) and Ge nano-dot formation procedure (b). **Due to the properties of low melting point and higher nucleation process of Ge, dots were self-assembled and formatted dots by the SK growth mode.

Figure 1 shows the localization of self-assembled Ge nano-dots produced by excimer laser annealing without SEG technique on a pre-patterned wafer. First, we deposited poly-Si with nominal thickness approximately 5 nm on a buffer oxide/Si substrate to reduce the incubation time for depositing Ge. Poly-Ge was then deposited with nominal thickness approximately 100 nm. All depositions were carried out in an UHV-CVD system with a base pressure of approximately 1 × 10^-9^ Torr. A pure GeH_4_ flow of 100 sccm was used as the gas precursor, with a growth temperature of 500°C. Figure 2a shows a top-view scanning electron microscope (SEM) of the as-deposited UHV-CVD poly-Ge, where a poly grain shape was clearly observed with a grain size of several hundred nanometers. The poly-Ge was irradiated by an excimer laser (XeCl) with 308-nm wavelength and 100-ns pulse duration to cause melting and re-crystallization. Due to the low melting point and higher nucleation density of Ge, dots were self-assembled and formed by the Stranski-Krastanov (SK) growth mode, as shown in Figure 2b. The well-known SK growth mode for the self-assembly of semiconductor crystals was initially demonstrated on III-V and II-VI families of semiconductor materials [12,13]. 

## Results and discussions

**Figure 3:**
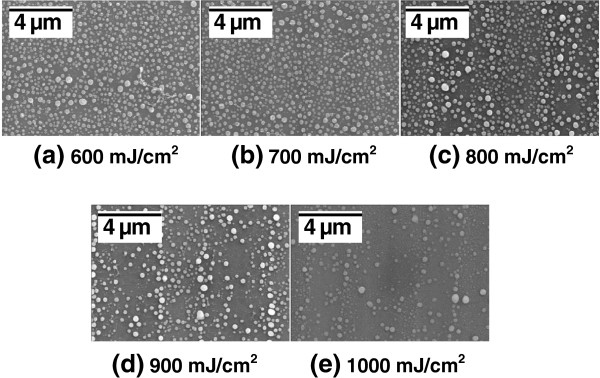
**The Ge dots distribution by top-view SEM. **The dots formation by nucleation is larger and sparse with laser energy increasing, and leads uniformity decreasing.

**Figure 4:**
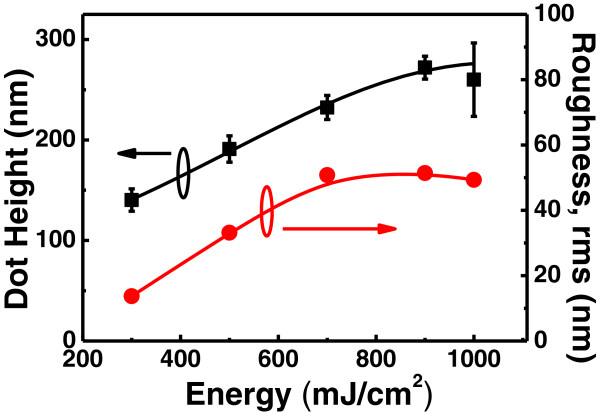
**The dot height and surface rms (root-mean-square) value vs laser energy. **The dot height (black colored line) and roughness (red-colored line) are increasing with higher laser energy, they saturated about 600 to 800 mJ/cm^2^.

**Figure 5:**
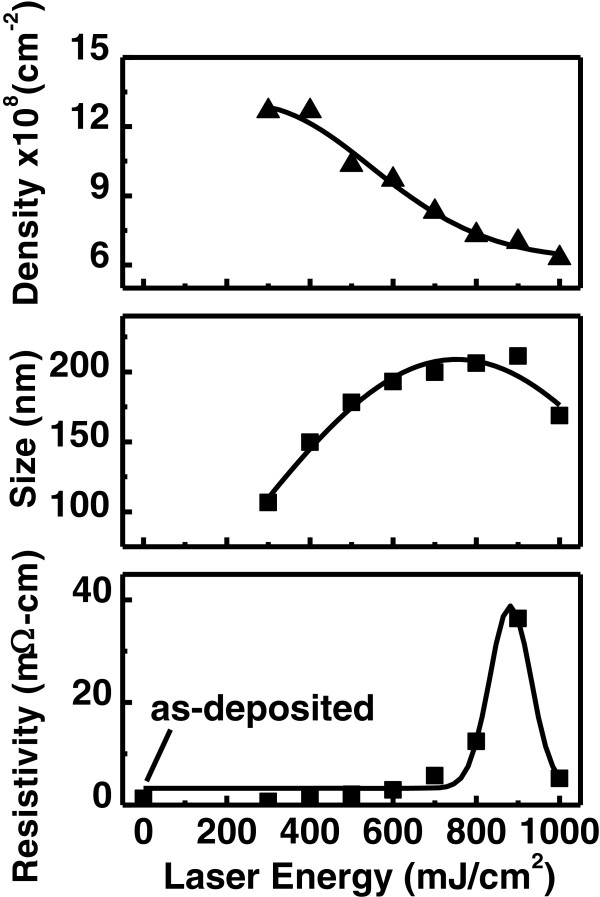
Ge nano-dots density, size, and resistivity vs. laser energy.

Figure 3 shows a top-view SEM of Ge self-assembled nano-dots after laser annealing with the energy from 600 to 1,000 mJ/cm^2^. A large and sparse dot distribution was obtained with increasing irradiation energy. Note that the buffer oxide underneath the Ge layer was ~approximately 500-nm thick; such thick oxide layers could assist heat retention during laser illumination due to a low thermal conductivity. The dot height and the surface root-mean-square (rms) value were obtained by cross-sectional high-resolution transmission electron microscope (HR-TEM) and atomic force microscopy respectively, as shown in Figure 4. In order to investigate the relationship between dot formation and laser energy, the statistical distribution of dot density and size was prepared by observing top-view SEM (scanning electron microscope), as shown in Figure 5. This revealed that denser dots with smaller sizes were created at lower laser energy. Due to the higher nucleation density of Ge, higher laser energy might have caused the melting of poly Ge and the mixing of adjacent Ge nucleation sites to form larger dots, which were self-assembled by the SK growth mode. This indicated the decreasing thickness of the Ge wetting layer, and the acquisition of higher sheet resistances by four-point probe measurement, as shown in Figure 5. Ge nano-dots by laser annealing method with the energy from 300 to 1,000-mJ/cm^2^ were fabricated, and the 0 mJ/cm^2^ meant as-deposited poly-Ge, which indicated no dots formation to obtain density and size. Note that the dot size had a maximum of about 200-nm diameters around the laser energy of 700 to 900 mJ/cm^2^, and the highest resistivity was obtained at the laser energy of 900 mJ/cm^2^. Higher laser energy (>900 mJ/cm^2^) might induce Ge melting and sublimation, and make island size and resistivity decrease. According to the size and density in Figure 5, the total volume of Ge would be decreased with higher laser energy (> 900 mJ/cm^2^). This indicates the partial Ge vapor and/or sublimation, and makes dot size and resistivity decrease. 

**Figure 6:**
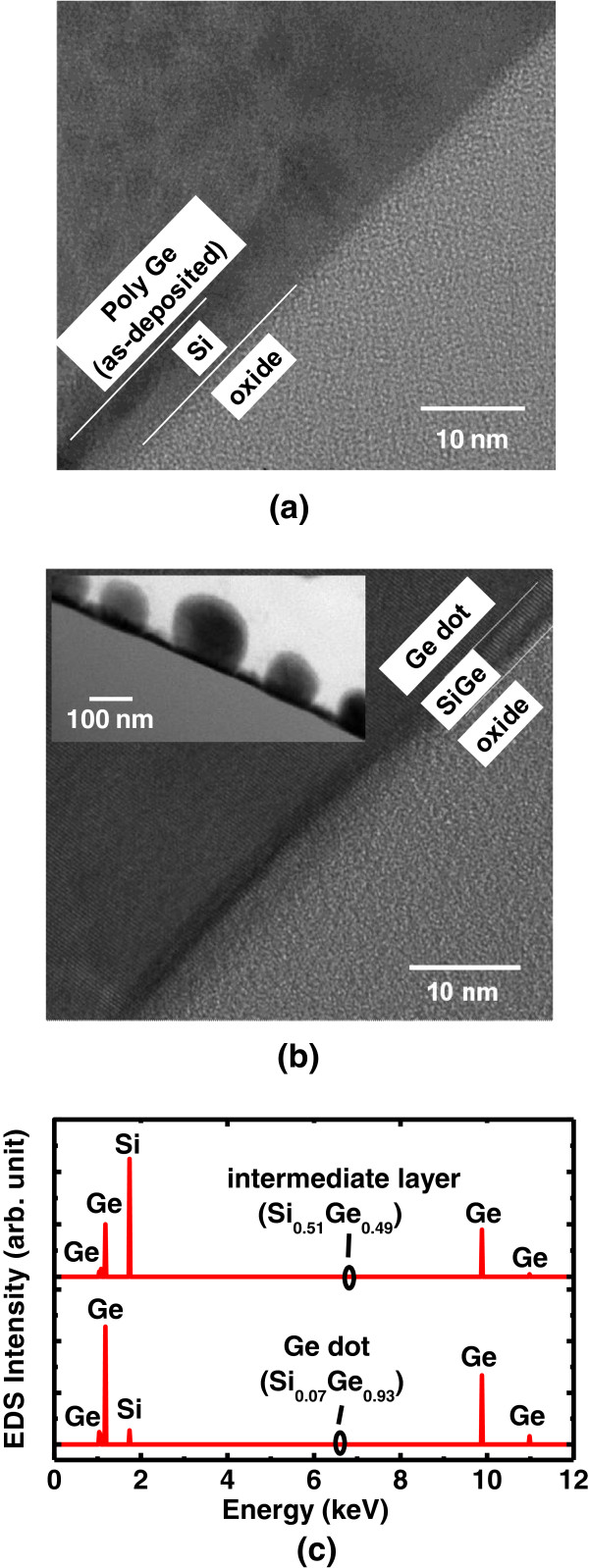
**The cross sectional HR-TEM images. **(**a**) as-deposited poly Ge and (**b**) Ge dot with laser energy of 700 mJ/cm^2^. The inset of (**b**) shows the cross-sectional TEM image of Ge dots. The crystallization quality of Ge dot is also improved as compare with as-deposited poly Ge. (**c**) The compositions of intermediate layer and dot by EDS after laser annealing. The Ge content of intermediate layer and dot are 49% and 93%, respectively.

**Figure 7:**
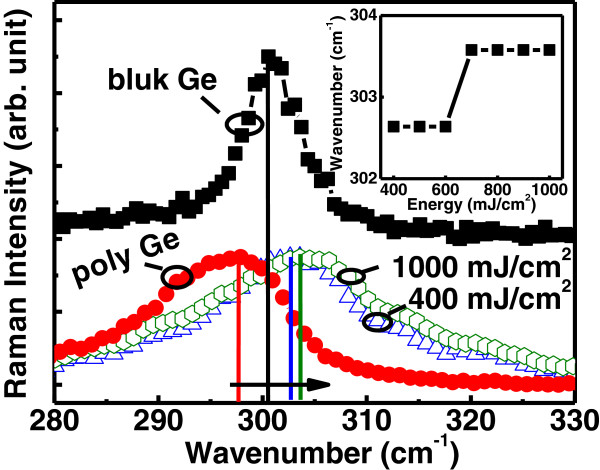
**The Raman spectra. **As-deposited poly Ge and Ge nano-dots with laser energy of 400 and 1,000 mJ/cm^2^. The bulk-Ge is for reference [14]. The inset shows the abrupt transition in strain which occurred between 600 to 700 mJ/cm^2^.

Cross-sectional HR-TEM images of the as-deposited poly-Ge and the dots with laser energy of 700 mJ/cm^2^ are shown in Figure 6a,b, respectively. The inset of Figure 6b shows the cross-sectional HR-TEM image of Ge dots. The Si layer was used for reducing Ge incubation time, which was approximately 6- and 4-nm thick for as-deposited and laser annealing with energy of 700 mJ/cm^2^, respectively. The compositions of intermediate layer and dot were confirmed by energy-dispersive X-ray spectroscopy (EDS) with the Ge contents of 49% and 93%, respectively, as shown in Figure 6c. This indicates the as-deposited intermediate layer Si and poly-Ge would be inter-diffused and inter-mixed to form SiGe at interface between dot and oxide. The Ge concentration was kept more nearly pure Ge in the dot with laser annealing. It was clearly observed that the crystallinity of Ge was improved with laser irradiation as compared with the as-deposited poly-Ge. The Raman spectra of the as-deposited poly-Ge and Ge nano-dots for laser energy of 400 and 1,000 mJ/cm^2^ are shown in Figure 7. The Ge-Ge phonon frequencies at Γ points were 297.9, 302.6, and 303.6 cm^−1^ for poly Ge and Ge nano-dots with laser energy of 400 and 1,000 mJ/cm^2^, respectively. Note that bulk Ge is for reference. As compared with bulk Ge (300.6 cm^−1^) [14], the Raman shift of the Ge-Ge phonon frequency (*Δω*_Ge-Ge_) of poly-Ge indicated tensile strain of 0.675%. A blue shift of the Ge-Ge peak for Ge nano-dots was observed, with the shift increasing for higher laser energy, indicating increased compressive strain. The smaller lattice constant of the nano-dots is due to the partial Si diffused and mixed into Ge to form SiGe with compressive strain after laser irradiated as shown in Figure 6. The inset of Figure 7 shows an abrupt transition which occurred between 600 and 700 mJ/cm^2^. With laser energy more than 700 mJ/cm^2^, the intermediate layer Si may fully melt and randomly nucleate the SiGe dots. This indicated smaller grain size formation in the dot and the compressive strain increasing with increment in surface energy due to larger grain surface area. After laser irradiation, Ge nano-dots sustained internal compressive strain, leading to a greater dot height and rough surface, as shown in Figure 4. 

## Conclusions

The selective pre-pattern free self-assembly of Ge nano-dots was demonstrated, and discussed as a function of laser energy. The advantage of this technique is patterning localization without SEG, reducing cost and improving throughput. Self-assembled Ge nano-dots were produced by excimer laser annealing, and the statistical distribution of dot density and size was collected for different laser energy. Improvement in crystallization quality of Ge dots was studied, and a strain analysis was carried out.

## Competing interests

Both authors declare that they have no competing interests.

## Authors' contributions

MHL created the idea and designed the study, carried out the sample fabrication, laser annealing, SEM, TEM, and electrical analysis, and drafted the manuscript. PGC participated in the EDS. Both authors read and approved the final manuscript.
